# Galectin-3 and Cyclin D3 Immunohistochemistry and Tumor Dimensions Are Useful in Distinguishing Follicular Oncocytic Carcinomas from Oncocytic Adenomas of the Thyroid

**DOI:** 10.1155/2015/276854

**Published:** 2015-10-29

**Authors:** C. Cacchi, H. M. Arnholdt, C. J. Haas, H. Kretsinger, L. Axt, B. Märkl

**Affiliations:** ^1^Institute for Pathology, Uniklinik RWTH Aachen, 52074 Aachen, Germany; ^2^Institute for Pathology, Klinikum Augsburg, 86156 Augsburg, Germany; ^3^Clinic for General, Visceral and Transplantation Surgery, Klinikum Augsburg, 86156 Augsburg, Germany

## Abstract

*Aims*. Oncocytic (Hurthle) follicular cell tumors (OTs) of the thyroid are both adenomas (OAs) and follicular carcinomas (OCs). The routine diagnosis of these tumors can be problematic even after an accurate sampling and histological examination. Beside preoperative evaluation due to the tumor's dimension several studies have been performed to find markers able to distinguish malignant from benign follicular tumors in the thyroid, with Galectin-3 being one of the most effective. Recently, some authors suggested cyclin D3 as adjunct to the diagnosis of the oncocytic lesions of the thyroid. *Methods and Results*. In this paper we assess the role of Galectin-3 and cyclin D3 in a well-selected group of follicular oncocytic tumors (14 OCs and 26 OAs). The diameter of each lesion was also evaluated. The combination of Galectin-3 and cyclin D3 has a good specificity (81%) and sensitivity (100%). Moreover, the maximum diameter (in cm) of OCs is greater than OAs (4.1 versus 2.3).* Conclusions*. We believe that the use of Galectin-3 and cyclin D3 in OTs of the thyroid can be a helpful panel in daily practice when histology is doubtful.

## 1. Introduction

Hurthle cell thyroid tumors (also called oncocytic or oxyphilic thyroid tumors) are uncommon thyroid lesions. By definition, they are composed predominantly of follicular cells (75% or more) with deeply eosinophilic cytoplasm on haematoxylin and eosin (H&E) stained sections [1]. In the past [2], it has been proposed that all Hurthle cell neoplasms should be regarded as malignant or potentially malignant.

Recently, these tumors have been divided into two categories: the benign oncocytic adenoma (OA) and its malignant counterpart, follicular oncocytic carcinoma (OC), showing blood vessel invasion and/or capsular penetration. OC is an uncommon thyroid malignancy, representing about 2-3% of all thyroid carcinomas [3]. Some tumors may also have relatively small cells, high nucleus/cytoplasm ratios, and solid/trabecular architecture. For such lesions, having one or more of these features but lacking unequivocal sign of malignancy, Rosai and Tallini have suggested the following terms: atypical Hurthle cell adenomas/Hurthle cell tumors of uncertain malignant potential (HCT-UMP) [1]. Rosai and the Chernobyl thyroid group of pathologists recommended, for tumors showing questionable capsular invasion, the term follicular tumor of uncertain malignant potential (FT-UMP) if papillary thyroid carcinoma- (PTC-) type nuclear changes are absent and well differentiated tumor of uncertain malignant potential (WDT-UMP) in case of questionable (incomplete) PTC-type nuclear changes [1]. Several attempts have been made to support the distinction between follicular adenomas and carcinomas, including the oncocytic type, using clinical characteristics and immunohistochemistry. Sippel et al. [4] found an association between tumor dimensions and risk of nodal positivity in patients diagnosed with an oncocytic thyroid tumor by preoperative fine needle aspiration (FNA) biopsy. Similarly, Erickson et al. [5] showed that the larger the size of an oncocytic lesion, the higher the risk of malignancy.

Many markers have been used to classify oncocytic neoplasms. Among these, Galectin-3 (Gal-3), a *β*-galactoside-binding protein involved in regulating cell-cell and cell-matrix interactions, is a very promising one and has been shown to represent a very helpful adjunct to FNA biopsy of thyroid nodules [6]. Regarding Hurthle cell thyroid lesions, some authors [7] have demonstrated a very good sensitivity (95.1%) and specificity (88%) for Gal-3 as a marker for OCs. Furthermore, in combination with the marker HMBE-1, they reported an excellent sensitivity for this panel (99%). However, other papers [8–10] have suggested that Gal-3 immunoreactivity is not restricted to malignant neoplasms but can also be detected in thyroid adenomas.

Some investigators [5–11] have performed immunohistochemistry studies focusing their attention on proliferative activity (Ki-67) and found lower activity in OAs compared to OCs. Moreover, other results [12] have shown that high proliferative activity is evident only in Hurthle cell carcinomas with clinically aggressive behaviour. Recently, studies have been performed to assess the role and diagnostic value of D-cyclins as diagnostic markers in cases of oncocytic thyroid lesions. The cyclin D family plays a pivotal role in the regulation of G1/S-phase cell transition [13], and D-cyclins are overexpressed in different types of cancers [13]. Cyclin D1, for example, was found to be absent in normal thyroid tissue [12] and was as effective as cyclin D3 (100% specificity) in determining the behaviour of FNA specimens suspicious for Hurthle cell neoplasia [14]. Both of the markers, however, showed a low sensitivity; thus, the authors conclude that there should be increased suspicion for malignancy in indeterminate oncocytic lesions of the thyroid that overexpress cyclin D3. They concluded that the risk of malignant behaviour increases with the rate of cyclin D3-expressing cells. Its diagnostic value is improved by combination with cyclin D1 evaluation.

Troncone et al. [15] demonstrated that, in OCs, the accumulation of p27 (kip1) is associated with cyclin D3 overexpression, suggesting that cyclin D3 is a helpful marker when the histology is unclear. The aim of our study was to evaluate the hypothesis that a combination of tumor diameter and immunohistochemical expression of cyclin D3 and Gal-3 is helpful in distinguishing between benign and malignant thyroid lesions.

## 2. Material and Methods

We retrieved all cases of thyroid specimens from 1995 to 2007 matching the key words: “Oncocytic OR Oxyphilic OR Hurthle”. All cases of medullary and papillary carcinomas (oncocytic variants) were excluded. After registering the diameter of each specimen in the initial cohort of 96 cases, we excluded cases of hyperplastic nodules and oncocytic metaplasia and cases intermingled with poorly differentiated carcinomas. All slides of the remaining 58 cases were reevaluated by investigator Claudio Cacchi (CC). Only cases that clearly had capsular and/or vascular invasion (OCs) and cases that clearly did not have vascular and/or capsular invasion (OAs) were chosen for further investigation. We obtained a final group of 40 cases (14 OCs and 26 OAs). Formalin-fixed, paraffin-embedded tissue specimens were available from each case. A representative sample of each lesion was selected for the successive immunohistochemical investigation. 3-4 *μ*m slides were cut and stained for Gal-3 (Monoclonal Mouse Anti-Human; Clone 9C4, dilution 1 : 100, Novocastra) and for cyclin D3 (Monoclonal Mouse Anti-Human; Clone DCS-22, dilution 1 : 40, Novocastra) to assess the percentage of positive tumor cells. Immunostains were developed according to an antigen retrieval treatment (in citrate buffer at pH 6.0) using a biotin-free detection system (En Vision, DakoCytomation).

Each antibody was tested with external positive and negative control, and for Gal-3 we also considered foamy macrophages to be an internal positive control. Both Gal-3 and cyclin D3 expression were assessed/evaluated by two independent investigators Claudio Cacchi (CC) and Bruno Märkl (BM), without knowledge of the clinic pathological status of the cases. Each slide was scanned at low power magnification (×1.6 lens) to assess the average percentage of tumor cells positive for each marker. We considered true positive cells to be only those that showed nuclear reactivity for cyclin D3 and concomitant/simultaneous nuclear and cytoplasmic reactivity for Gal-3. For Gal-3 and cyclin D3, cutoffs of ≥1% and ≥25% have been chosen to classify cases as positive [7, 14].

Results from investigator Claudio Cacchi (CC) were used to evaluate the specificity and sensitivity of each antibody tested alone or in combination.

This study was performed according to the national rule, that is, law of hospitals in Bavaria (Bayerisches Krankenhausgesetz). This study has been also examined by the internal review board.

## 3. Statistics

For the comparison of the results, the paired *t*-test or Mann-Whitney rank sum test was used, depending on the normality test. *P* values < 0.05 were considered significant.

The averaged results are shown as mean values ±1 standard deviation (SD). All calculations were performed using the Sigma Plot 11.0 software package (Systat, Richmond, USA).

A kappa value (*κ*) was also calculated to estimate the interobserver agreement for both immunohistochemical evaluations; an absolute difference of ≤10% in two measures was considered agreement.

## 4. Results

The maximum diameter (in cm) of OCs was greater than in OAs (4.1 ± 2.3 versus 2 ± 0.8) (*P* < 0.001).

The results of the investigators had good [16] agreement for both Gal-3 and cyclin D3 (*κ* = 0.8 and 0.7, resp.).

**Figure 1:**
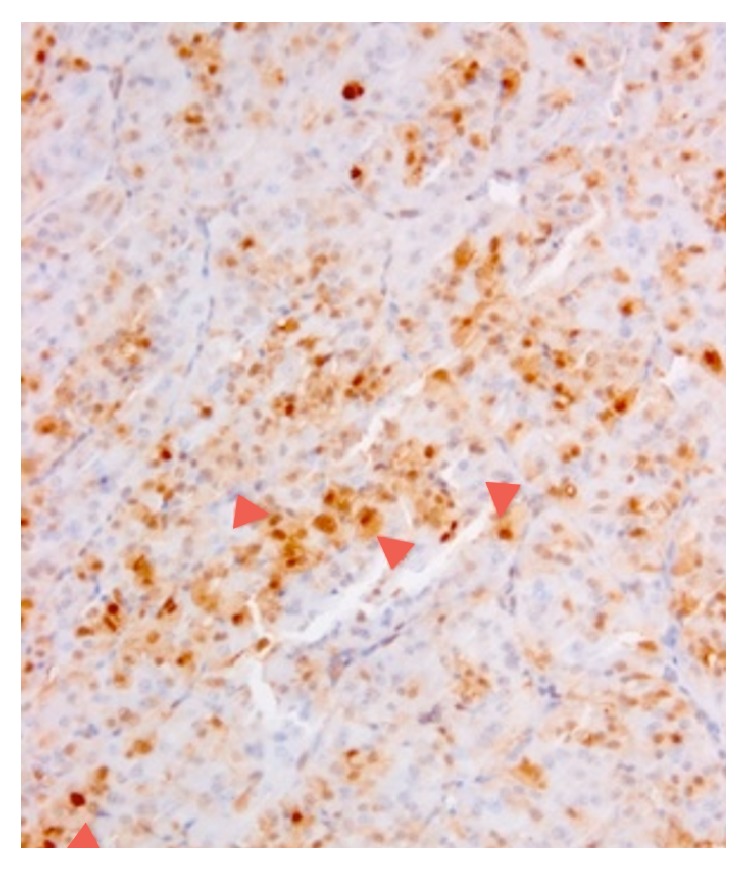
Immunohistochemical features. It should be noted that also Hashimoto thyroiditis may show reactivity for Galectin-3, but it is typical focal and restricted to epithelial cells in the areas of lymphocytic infiltrations. Gal-3 in OC: note the diffuse reaction in tumor cells (arrowheads).

**Figure 2:**
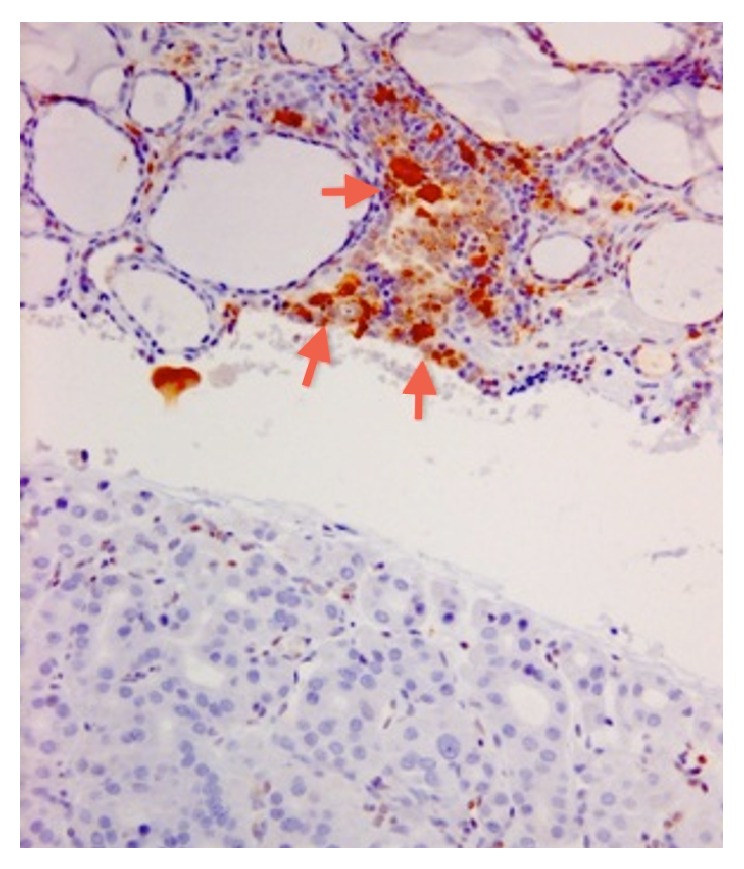
Immunohistochemical features. It should be noted that also Hashimoto thyroiditis may show reactivity for Galectin-3, but it is typical focal and restricted to epithelial cells in the areas of lymphocytic infiltrations. Gal-3 in OA: compare the negative tumor with the positive macrophages (top, arrows).

The mean values of Gal-3 in OCs versus OAs were (Claudio Cacchi (CC) and Bruno Märkl (BM)) 25% ± 25% and 24% ± 26% versus 3% ± 8% and 6% ± 17% (Figures 1 and 2). The examiners' results (Claudio Cacchi (CC) and Bruno Märkl (BM)) showed a significant difference in percentage (*P* value < 0.001 and 0.002) of positive tumor cells between OCs and OAs.

**Figure 3:**
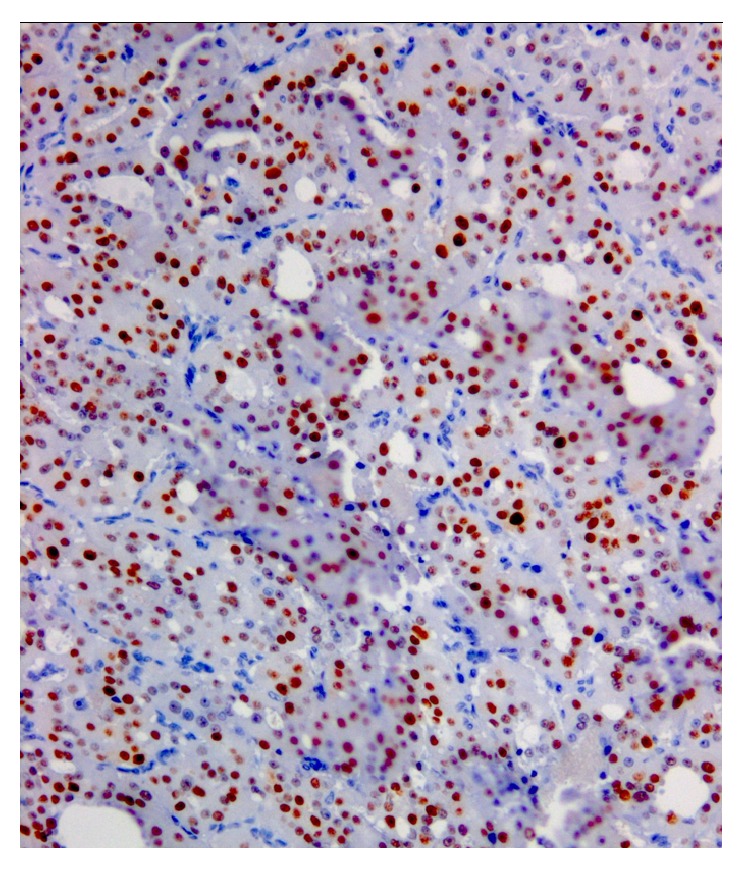
Immunohistochemical features. It should be noted that also Hashimoto thyroiditis may show reactivity for Galectin-3, but it is typical focal and restricted to epithelial cells in the areas of lymphocytic infiltrations. Immunostaining with cyclin D3 with a diffuse and strong reaction in OC.

**Figure 4:**
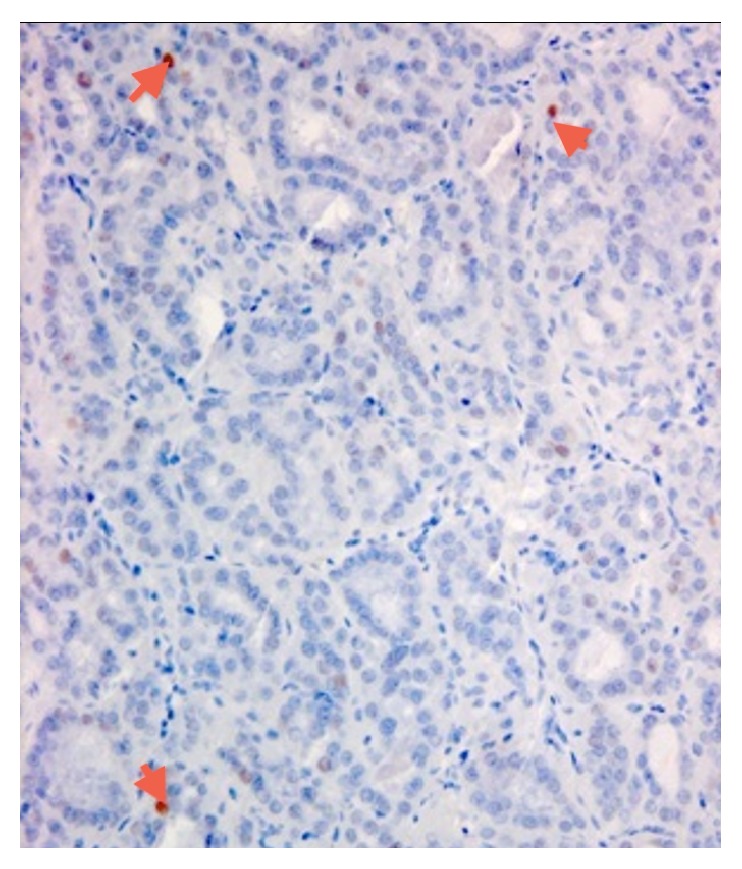
Immunohistochemical features. It should be noted that also Hashimoto thyroiditis may show reactivity for Galectin-3, but it is typical focal and restricted to epithelial cells in the areas of lymphocytic infiltrations. OA: only few cells show a weak positivity for cyclin D3 (arrows).

The results for cyclin D3 are the following (Claudio Cacchi (CC) and Bruno Märkl (BM)): 46% ± 37% and 47% ± 33% in OCs and 8% ± 13% and 13% ± 17% in OAs (Figures 3 and 4). The two evaluations also showed cyclin D3 overexpression in the OCs with respect to the OAs (*P* < 0.001 and *P* = 0.001, resp.).

Gal-3 as marker of malignancy showed a relatively good sensitivity (79%) and specificity (81%). Although the sensitivity of cyclin D3 as a single marker was low (50%), its specificity was very high (96%). A combination of the two markers (Gal-3+ OR cyclin D3+) demonstrated excellent sensitivity (100%) and a quite good specificity (81%). The results are summarized in Tables 1 and 2.

## 5. Discussion

For the FT-UMP or the HCT-UMP cases, Papotti et al. [17] demonstrated that there is no benefit with Gal-3 and HBME-1 immunoprofiling. Herein we studied only cases with either certain benignity or certain malignancy.

Regarding tumor dimensions, we described an important difference in the mean diameter of OCs (4.1 cm) in comparison to OAs (2 cm). These findings are similar to data found in the literature. In fact, Erickson et al. [5] reported a median diameter of 4.8 cm for OCs and 3.1 cm for OAs. Furthermore, Sippel et al. [4] demonstrated that OCs are larger than OAs (5.0 versus 2.7 cm), supporting the predictive role of neoplasm's dimensions in cases of FNA biopsy with examination of oncocyte cytoplasm. In these cases [4], no malignancy was found for lesions smaller than 2 cm. In our study, only two out of fourteen cases (14%) in the OC group were <2 cm (1.5 and 1.7 cm).

Our results, in agreement with the literature [4, 5], underscore the importance of clinical information and/or macroscopic precision in such lesions. The correlation between tumor diameter and risk of malignancy of oncocytic tumors of the thyroid suggests the hypothesis of time-related carcinogenesis, in which an oncocytic adenoma represents a premalignant lesion, and the FT-UMP represents “formae frustrae” of carcinomas. The results published by Erickson et al. [5] seem to support this hypothesis, showing a diameter of 3.7 cm for neoplasms of uncertain malignant behaviour (UMB), which is larger than adenomas but smaller than carcinomas.

The use of Galectin-3 as an adjunct to routine diagnosis of follicular thyroid neoplasms has been previously demonstrated [6, 18, 19]. However, other studies have found reactivity for Gal-3 in adenomas [8–10]. A possible explanation for these discrepancies may be the presence of false positive reactivity for Gal-3 due to biotin dependent detection systems. For this reason, we used a biotin-free system (En Vision, DakoCytomation).

Focusing on oncocytic cell tumors of the thyroid, Volante et al. showed a 94.3% rate of Gal-3 positive carcinomas and a 12% rate of Gal-3 positive adenomas. In this study, Gal-3 had a sensitivity of 95.1% and a specificity of 88%, and if used in combination with HBME-1, the sensitivity was 99%. Interestingly, another study [8] illustrated a significant difference in immunostaining for Gal-3 between OCs and OAs (59% versus 7.1%). This diversity of percentage in Gal-3 immunostaining in our study is similar to the literature; however, probably due to the relatively small number of carcinomas in our study, the specificity and sensitivity for Gal-3 are lower than those previously reported [7].

Factors that affect cell cycle machinery, recently well elucidated, also have been tested in oncocytic follicular thyroid lesions.

For example, Müller-Höcker [11] showed a higher reactivity for p53 in oncocytic neoplasms of the thyroid (88% in OCs and 75% in OAs; eight of seventeen OCs but only three of twenty OAs with reactivity in more than 10% of the cells) and showed a higher cell proliferation in OCs than OAs (Ki-67: 76 and 12 cells per 10/HPF, resp.). Hoos et al. suggested, on the basis of a higher Ki-67 index in widely invasive Hurthle cell carcinoma, that this marker may have a role in the diagnosis of Hurthle cell thyroid tumors. In 2000 [5], Erickson et al. proposed Ki-67 and cyclin D1 as helpful indicators in distinguishing oncocytic adenomas from carcinomas.

Recently, a study performed on FNA samples with oncocytic features using cyclin D3 and cyclin D1 as predictors of malignancy illustrated a very good specificity (100%) but low sensitivity (32 and 79%, resp.). The authors of this study [14] adopted cutoffs of 6.5% and 7.5% to improve the predictive value of cyclin D1 and cyclin D3, respectively, and they recommend combining the two markers to enhance this capability. Interestingly, Troncone et al. demonstrated that increased p27 expression in oncocytic follicular carcinoma is a consequence of cyclin D3 overexpression and suggested that cyclin D3 was a valid immunohistochemical marker for distinguishing OCs from OAs in cases of unclear histology. Although translocation *t*(6; 14)(p21.1; 32.3) or amplification of the cyclin D3 gene is observed in different neoplasms, the mechanism of the overexpression of cyclin D3 in OCs is still unclear and further investigation in this direction is needed.

Our study is the first that combines dimension, a nuclear marker (cyclin D3), and a nuclear-cytoplasmic marker (Gal-3) to differentiate oncocytic carcinomas and adenomas. Because of the possibility of a focal reaction for Gal-3 in adenomas and to avoid the risk of using a very low threshold value, we did not use a cutoff in evaluation of this reaction.

On the other hand, we believe that adopting a cutoff of 25% for cyclin D3 is quite easy to perform and reduces the interobserver variability in routine practice.

Our results on the cyclin D3 expression in tumor cells are in accordance with those shown by Troncone et al., with a low interobserver variability (*κ* = 0.7).

In conclusion, our study, which was performed in a well-selected series of follicular oncocytic thyroid tumors, indicates that the combination of Gal-3 and cyclin D3 has excellent sensitivity (100%) and a relative good specificity (81%), it is easy to perform, and it demonstrates a good interobserver agreement.

Moreover, we believe that this panel also may be a useful combination in FNA cytology or cytological cell-block material, because oncocytic follicular lesions of the thyroid could represent a diagnostic challenge in the praxis. Moreover, it is important to improve the management of these patients, in order to avoid unnecessary operations [20]. Positive results of this panel in case of histological morphology of adenoma may indicate performing additional section to exclude a capsular or vascular invasion.

It also could be of interest to evaluate this same panel in oncocytic follicular tumors of uncertain malignant potential, after a proper follow-up, to understand better the behaviour of these lesions.

## Figures and Tables

**Table 1:** 

	Oncocytic carcinomas (14)	Oncocytic adenomas (26)
Diameter (cm)	4.11 ± 2.3	2 ± 0.8
Cyclin D3 (Claudio Cacchi (CC)) (% of tumor cells)	46 ± 37	8 ± 13
Cyclin D3 (Bruno Märkl (BM)) (% of tumor cells)	47 ± 33	13 ± 17
Galectin-3 (Claudio Cacchi (CC)) (% of tumor cells)	25 ± 25	8 ± 13
Galectin-3 (Bruno Märkl (BM)) (% of tumor cells)	24 ± 26	13 ± 17

**Table 2:** 

	Cyclin D3	Galectin-3	Gal-3 OR cyclin D3
Sensitivity	50%	79%	100%
Specificity	96%	81%	81%
